# E-cigarettes use in the United States: reasons for use, perceptions, and effects on health

**DOI:** 10.1186/s12889-020-09572-x

**Published:** 2020-10-09

**Authors:** Sakshi Sapru, Mridula Vardhan, Qianhao Li, Yuqi Guo, Xin Li, Deepak Saxena

**Affiliations:** 1 Department of Molecular Pathobiology, New York University College of Dentistry, 345 E 24th St, Room 921B, New York, NY 10010 USA; 2 Departments of Urology, New York University School of Medicine, New York, NY 10016 USA; 3 Departments of Surgery, New York University School of Medicine, New York, NY 10016 USA

**Keywords:** E-cigarettes, Nicotine, Aerosol, Vaping, Flavoring agents, Smoking, Oral heath, Cardiovascular health

## Abstract

**Background:**

Many researchers claim electronic cigarettes (e-cigarettes) to be a breakthrough invention for tobacco users that aspires to curb their addiction to conventional cigarettes. Claimed to be safer by their promoters, these smokeless devices have become increasingly popular since their arrival on the market among users of all ages, especially adolescents. This paper investigated the trends in e-cigarette usage since the time it arrived in the United States, highlighting the highest surge that has occurred in adolescent e-cigarette use. It also aimed to understand the reasons and perceptions behind the ever-increasing use of e-cigarettes by adolescents.

**Main body:**

With the advent of e-cigarettes and common positive perceptions regarding their use, we are at risk of reversing the years of efforts regarding tobacco control and instead advance towards a new addiction with currently unknown long-term health hazards. There is substantial data showing a significant increase of e-cigarette users in the United States, especially among adolescents. The aim of this review was to explore the reasons behind this widespread increase in the use of e-cigarettes among the teenage population in the US and also to uncover the common perceptions about these new electronic delivery systems. In addition, this review attempted to summarize health benefits and hazards associated with e-cigarette use as it crucial to have the right information among its users regarding the health effects of e-cigarette use.

**Conclusion:**

E-cigarettes are more appealing than c-cigarettes for a variety of reasons, including cost, choice of different flavors, ease of accessibility, and use and impact of social media. There are also different perceptions among e-cigarette users, including both adolescents and adults. The former group may use them because of the sense of fashion associated with this novel device, and the latter might intend to quit conventional/combustible cigarettes (c-cigarettes) by switching to e-cigarettes. However, it is important to note that e-cigarettes are a recent phenomenon; therefore, there is a lack of many long-term studies that can identify future health risks associated with e-cigarette use. We need more detailed studies that focus on the long-term health effects of e-cigarette use. Moreover, with the ever-increasing usage of e-cigarettes by adolescents (10 and 19 years), it is very important that e-cigarettes be incorporated into the current tobacco-free laws and ordinances. We conclude by stating that e-cigarettes need stronger regulations to prevent youth access and use.

## Background

The overall trend in e-cigarette smoking among adolescents (middle school [10–14 years] and high school [14–18 years]) students is higher than in the adult population. The number of current adolescent e-cigarette users has experienced an upward trend since e-cigarettes first arrived in the United States [1–3]. According to a US Health and Human Services report in 2017, a nonlinear increase for current use of e-cigarettes among high school students from 1.5 to 11.3% between 2011 and 2017 occurred. In the case of middle school students, the current use of e-cigarettes exhibited an upward progression from 0.6 to 4.3% between 2011 and 2017 [4]. The 2018 National Youth Tobacco Survey (NYTS) report stated that 3.6 million adolescents in the US were currently using e-cigarettes, and e-cigarette use has drastically increased, especially in the last few years (2017 to 2018) [5].

**Fig. 1:**
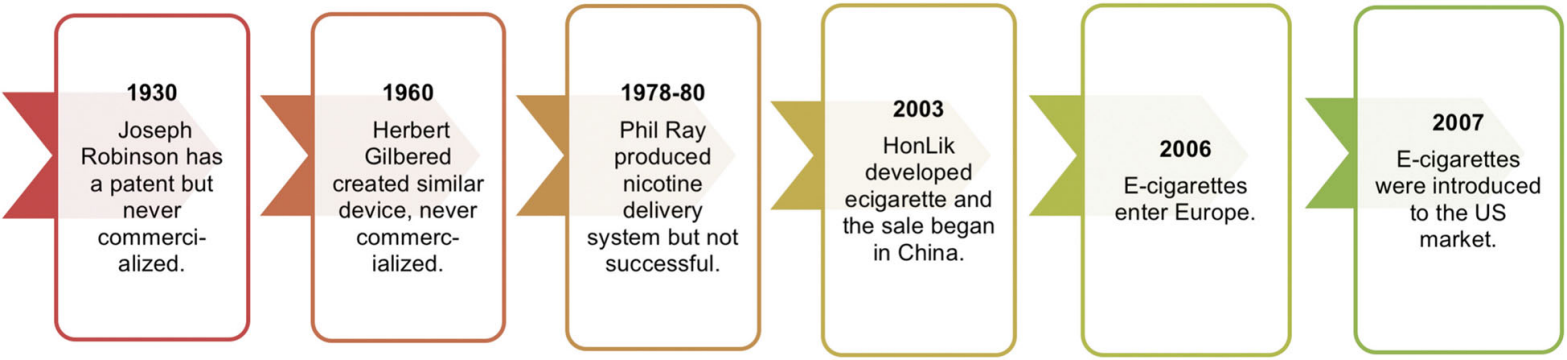
Timeline of electronic cigarettes (e-cigarettes)

**Fig. 2:**
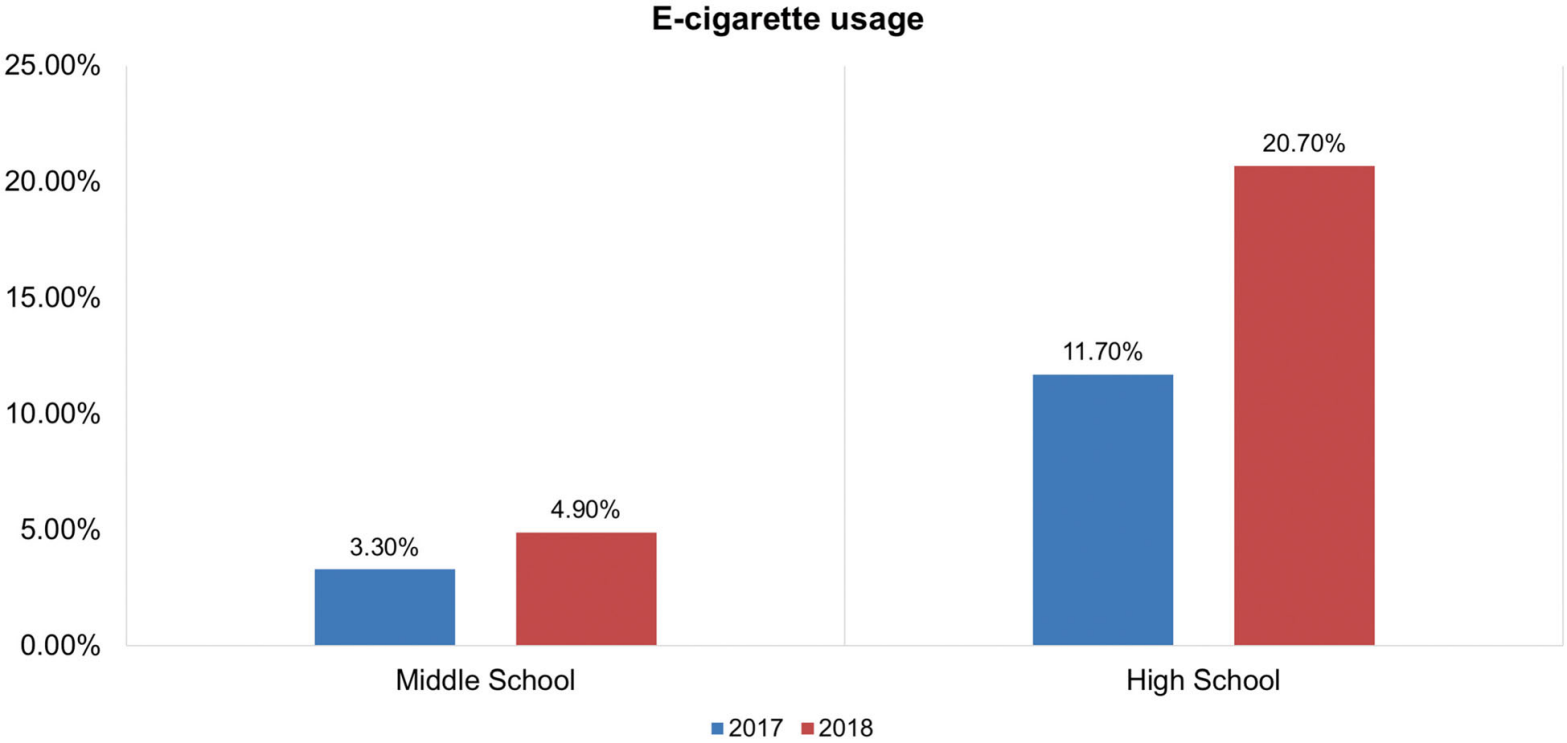
Trend of e-cigarette usage among United States adolescents in the year 2017–2018

Figure 1 illustrates the time line of e-cigarette introduction in USA and Fig. 2 depicts the “alarming increase” in the number of middle and high school e-cigarette users from 2017 to 2018. In the year 2018, CNN called e-cigarettes a “Teen Epidemic” in a news bulletin. The problem of adolescent e-cigarette use has become a great concern, and it is therefore important to investigate the adoption of electronic substitutes by teens [3]. This article investigated perceptions of e-cigarettes and further probes the reasons why e-cigarettes have become popular among users, especially adolescents.

### A brief History of E-cigarettes

A Chinese pharmacist named Hon Lik, in association with the company Dragon Holding, invented e-cigarettes, which they called Ruyan, meaning “smoke like”. The death of his father, who was a heavy smoker and died of lung cancer, motivated him to develop a less harmful alternative to c-cigarettes. E-cigarettes came onto the market in 2003 and were patented internationally in the year 2007. E-cigarettes entered the US market in the same year and since then, have gained popularity among both cigarette smokers and non-smokers (Fig. 1).

### Data concerning e-cigarette smoking trends over the years in adults and young adults

#### Adult population (> 25 years)

Among US adults, the number of e-cigarettes users increased from 1.8% in 2009 to 3.4% in 2010 [6]. The use of these products continued to increase in 2011 with 5.8% of U.S. adults reporting the use of e-cigarettes in that year [6]. Data from National Center for Health Statistics (NCHS) in 2015 showed that 3.7% of the U.S. adult population were current e-cigarette smokers. According to National Health Interview Survey of the United States in 2017, approximately 2.8% of the U.S. adult population were current e-cigarette smokers. According to Reuters Health, one in every 20 US adults has tried e-cigarettes. At present, approximately 10.8 million American adults use e-cigarettes [7]. Therefore, it is interesting to note that the number of current adult e-cigarette smokers showed an upward trend from 2010 until 2014 [8] followed by a decline in 2017 [9].

#### Young adult population (18–25 years)

According to the National Health Survey, the percentage of young adults who use e-cigarettes daily or some days increased from 2.4% in 2012 to 2013 to 5.2% in 2015. In addition, 40% of young adults who use e-cigarettes daily or some days were never smokers before trying e-cigarettes. In comparison with adults aged 25 years and older, young adults (18–25 years) are more likely to try e-cigarettes and report having used e-cigarettes in the past 30 days. Hence, we can see an increase in the number of e-cigarette users over the years among young adults aged 18 to 24 years.

#### Description of e-cigarettes and how they vary from c-cigarettes

Research has provided substantial evidence that control of nicotine addiction is beneficial for the health of smokers regardless of age. Over the years, tobacco control programs and interventions have demonstrated much success in decreasing initiation in non-smokers and cessation in smokers. However e-cigarettes many reverse this success in smoking cessation.

E-cigarettes are popular battery-operated devices used as an alternative for c-cigarettes. E-cigarette devices produce aerosol (gaseous mist) from a nicotine-containing liquid (e-liquid) and a heating element. The user then inhales the aerosol through the mouth and lungs from which it is absorbed into the bloodstream, and the remaining aerosol is exhaled. Many common terms are used for this device other than e-cigarettes, such as Juul (resembles a USB flash drive), e-vaporizer, vapes, electronic nicotine delivery systems (ENDS), and e-hookahs. E-cigarettes come in different shapes, including pen and regular cigarette shapes, portable pocket device, pen vaporizer, and box mod. There are two main types of e-cigarettes: (1) disposable and (2) cartridge models. The disposable type is an inexpensive alternative for beginners. On the other hand, the cartridge models are rechargeable and contain cartridges that can be refilled or exchanged.

E-cigarettes differ from c-cigarettes in their basic design, nature of the smoke, and the way the vapor is produced. The basic components of e-cigarettes include an aerosol generator, a flow sensor, a battery, and a nicotine-containing solution storage area. The effectiveness of e-cigarettes as a nicotine delivery system can be controlled with interchangeable parts, enabling users to modify the character of the delivered aerosol by changing the voltage [10]. E-cigarettes produce a gaseous substance/mist at a temperature that is lower than its point of combustion by heating the liquid in the cartridge, which consists of nicotine, propylene glycol, glycerol, or a mixture of water, flavor, and other toxic substances. Research has indicated the presence of trace levels of toxic compounds, namely, tobacco-specific nitrosamines (TSNAs), carbonyl compounds [11], and polycyclic aromatic hydrocarbons (PAHs) [12] in the e-liquids or aerosols of some commercially available products although most were found at much lower levels than in c-cigarette smoke. On the other hand, c-cigarettes produce smoke by burning tobacco with fire. This smoke consists of recognized carcinogens, such as the carbonyl compound, formaldehyde, organic compounds, such as benzene, tobacco-specific nitrosamines, free radicals, toxic gases, and heavy metals. There are more than 7000 chemicals present in c-cigarette smoke, among which 70 have been recognized as carcinogens [13, 14].

### What makes e-cigarettes more popular than c-cigarettes?

#### Reasons for choosing and perceptions of e-cigarettes among adolescent and adult users

According to research, various reasons have been reported by regular e-cigarette users and occasional users for why e-cigarettes might be more appealing than regular combustible cigarettes. Curiosity, smoking cessation, health benefits, and use by a friend or family member were a few common reasons apart from others, such as cost, easy availability, and convenience of use for starting e-cigarette use. According to one study [15], in which the reasons (self-reported) for trying e-cigarettes by adolescents were listed, curiosity was the top reason followed by good flavors, use by their friends and family, and finally low cost. In the study, some users claimed to have tried e-cigarettes because they thought that they were healthier than regular cigarettes, but only 5.9% tried to use them as a means to quit regular smoking. In another study [16] that explored the reasons for e-cigarette smoking by adults, the most often reported reason was smoking cessation followed by the health benefits of e-cigarettes over c-cigarettes. However, the study highlights that the appeal of e-cigarettes extended beyond smoking cessation. Some other reasons are described subsequently.

#### Cost effective

The average cost of a pack of c-cigarettes in the US in 2015 was $7.26; thus, the average cost of smoking c-cigarettes at a rate of one pack a day for one year equaled $2650 [17]. On the other hand, the average cost of smoking e-cigarettes (cartridge models) at the same rate was approximately $1000. Moreover, e-liquid is even cheaper to use with an average cost of $511 per year. Therefore, e-cigarettes are much more cost effective than c-cigarettes.

#### Multiple flavors

E-cigarettes come in more than one hundred flavors, which make them very attractive, especially for the younger generation. The availability of flavors such as candy-floss, cinnamon, chocolate, vanilla, bubble gum, and mint makes them desirable to everyone. According to research [18], these various flavor choices make e-cigarettes more appealing to adolescents over the tobacco-flavored c-cigarettes. Moreover, different flavors lead to smoking initiation [19] and smoking progression by masking the harsh taste of tobacco products, which is particularly appeal to young users [20]. Candy-, fruit- and menthol-flavored e-cigarettes appeal to adolescents more as compared to tobacco-flavored e-cigarettes [21].

#### Impact of social media

Social media also has a large influence on young people and has brought these electronic devices into the limelight. Many websites and web enthusiasts promote e-cigarettes, claiming them to be less harmful than combustible cigarettes and branding them as a healthier alternative. In 2012, the cigarette brand “Blu” was purchased by Lorillard Tobacco Company, which began airing commercials featuring celebrities using e-cigarettes. The sense of fashion and coolness portrayed by the models smoking e-cigarettes is also a big influence on young minds. Big tobacco companies have worked with organizations, such as the e-cigarette association, consumer advocates for smoke-free alternatives association, and Vapers International Inc., to delay or eliminate legislation aimed at limiting e-cigarette use and sales [22].

#### Ease of accessibility and use

E-cigarettes are available for online purchase with the ease of a click and can be widely seen in shopping mall kiosks, which is a common place for adolescents to spend much of their free time. This ease of purchase and availability has made e-cigarettes easily accessible to young students. Nonetheless, these vapes are easy to use anywhere because they do not leave behind the strong smell of tobacco and are easy to hide from adults. Kralikova and colleagues found that some people use e-cigarettes because they are allowed to use them in places in which the use of c-cigarettes is prohibited [23]. In a recent observational study, a potential concern has emerged due to placement of e-cigarettes near products that are popular with children in stores.

The American Academy of Pediatrics (AAP) has called for immediate federal intervention to restrict the marketing and sale of e-cigarettes to young people in its policy statement “E-cigarettes and similar devices” that was published in February 2019. The AAP also recommends that e-cigarettes be incorporated into the current tobacco-free laws and ordinances as stated in the AAP policy statement: E-cigarettes need stronger regulations to prevent youth access and use [24]. Nevertheless, the real question remains: *Are e-cigarettes a tobacco getaway or a nicotine trap?* Further research is essential for establishing a temporal relationship between e- and c-cigarette smoking. Can e-cigarettes be a helpful aid in cessation of c-cigarette smoking or does their convenience of use and other favorable factors attract more young non-smokers to try and adopt e-cigarettes and then eventually shifting to regular cigarette smoking?

### Common perceptions about E-cigarettes in users

Very little is known about the common perceptions, risks, or benefits associated with the use of e-cigarettes. It is very important to provide the right knowledge to users about the effects of these new nicotine delivery systems. Users may think that smoking e-cigarettes somewhat reduces the harmful effects of tobacco over e-cigarettes; however, they may not fully understand the overall effects of smoking e-cigarettes. Various studies indicate the potential for e-cigarettes to be misused as an entry point into regular tobacco use. According to one study, 9.3% of current e-cigarettes users in 2012 reported never having smoked c-cigarettes [25]. Therefore, it is understood that monitoring trends in user perceptions of absolute and relative harmfulness of tobacco products may provide an early warning of increasing acceptability and long-term use of e-cigarettes. According to various studies, some perceptions regarding the use of e-cigarettes in particular and relative to e-cigarettes exist:

- Adolescent users believe e-cigarettes are less harmful than c-cigarette smoking regardless of their previous smoking history, while others perceive e-cigarette to be harmful depending on the dose or the frequency of use [26]. In the same study, some users believe that they can control the adverse effects of e-cigarettes and quit smoking before becoming an addict. Because e-cigarettes are perceived to be less harmful, many smokers use e-cigarettes for goal-directed reasons, such as to quit regular cigarette smoking.
- A study including 9th to 12th grade students from California (mean age of 16 years) highlighted some of the common perceptions among users of this age group. The study found that 19.05% of participants believed that e-cigarettes contain water, 3.03% believed that e-cigarettes are not tobacco, 40.00% believed that e-cigarettes are useful for smoking cessation, and 43.13% believed that they are safer than c-cigarettes [27]. Overall, the participants believed that it is acceptable to use e-cigarettes both indoors and outdoors.
- One of the very common perceptions among adults is that their daily intake of caffeine from drinking coffee is comparable to the amount of nicotine they inhale from e-cigarettes. Since both of these products are used among working adults for the same reasons, such as feeling more energetic and alert and to improve concentration, people often inaccurately believe that their effects on health are similar [28–30]. The relationship between caffeine and nicotine is a growing debate in the public health community and is commonly discussed in terms of the substance proxies, that is, e-cigarettes and coffee. At present, there is a lack of comprehensive research comparing these two compounds.

### Effects of e-cigarettes on health

It is important to explore the composition of the e-liquid that is used in these electronic devices to understand the effects of e-cigarettes on health [1–3]. The main ingredient in the e-liquid is nicotine, which basically comes from tobacco. Other than nicotine, the e-liquid has a base, flavoring agents, and other chemicals. Several other alkaloids, such as nornicotine, anatabine, and anabasine are also indirectly added to the liquid via bacterial activity or oxidation during tobacco processing [31].

Many factors control the effects of e-cigarettes on health: (1) external factors (such as climate conditions, airflow, particulate size, number of users in the vicinity); (2) e-cigarette characteristics (such as type and age of the vaping instrument, battery voltage, puff length, interval between the puffs); and (3) user characteristics (such as age, gender, experience, health status of users) [32, 33].

E-cigarettes require greater effort to inhale the smoke than puffing a regular c-cigarette, and the amount and density of the aerosol decreases with time as the number of the puffs increases. In addition, reports say that the serum levels of nicotine are heterogeneous and vary with the user and the device [32]. A study of 16 different e-cigarettes reported that the nicotine level in the aerosol produced by 15 puffs ranged from 0.5 to 15.4 mg. On the other hand, in the smoke produced by conventional cigarettes, the typical levels are 1.54 to 2.60 mg [34, 35]. In this section, we summarize the overall effects of e-cigarettes on health.

### Benefits of E-cigarettes over traditional cigarettes

**Fig. 3:**
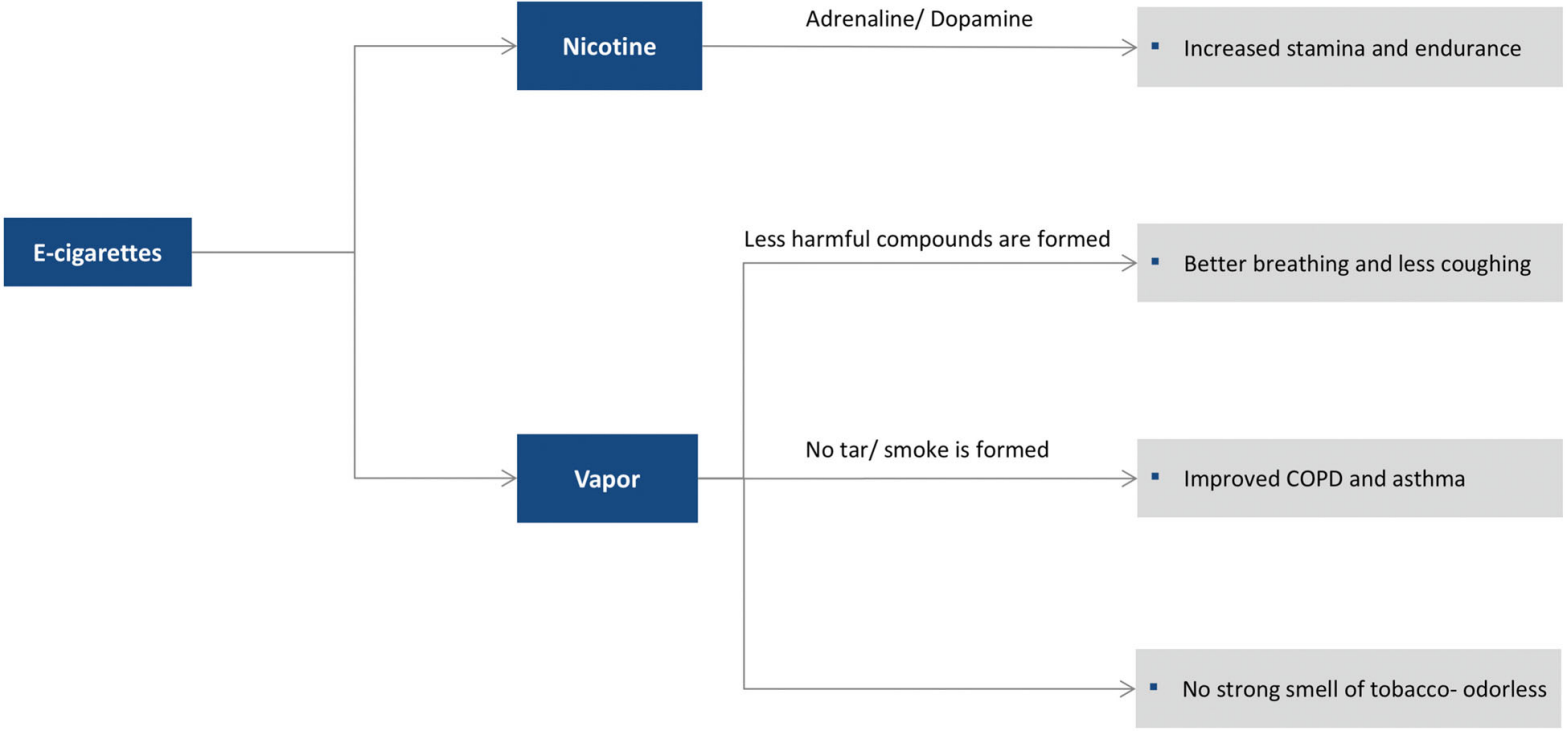
Benefits of using e-cigarettes versus the combustible cigarettes (c-cigarettes)

Many studies have shed light on the positive aspects of e-cigarettes, considering the less harmful health issues associated with e-cigarettes versus traditional c-cigarettes (Fig. 3).

#### Physiological effects

A case study by Polosa et al. addressing the health impact of e-cigarettes on daily users of e-cigarettes for a time period 3.5 years who had never smoked c-cigarettes highlighted some key findings [36]. The reference group consisted of 12 non-smokers with a mean age of 29.7 years. The aim of the study was to compare the health outcomes between e-cigarette users (previously non-smokers) and a group of non-smokers. This was the first study (conducted in the year 2013) that explored the health effects of prolonged exposure in a sample who did never smoked combustible cigarettes. They found no detectable changes in the lung health of the never smokers who smoked e-cigarettes regularly for nearly four years. In addition, there were no significant structural changes in the lungs or respiratory system. There were also no reports of any significant changes in systolic and diastolic blood pressure (BP) or heart rate, and no adverse cardiovascular effects in those smoked e-cigarettes regularly were noted. The major limitation of the study was the lack of long-term follow-up with the sample.

#### Respiratory effects

Studies of healthy smokers and smokers with asthma and chronic obstructive pulmonary disease (COPD) who switched to e-cigarettes have not shown any clinically significant adverse respiratory effects; however, they have actually shown a change in the harmful effects of smoked tobacco on the lung [37]. In addition, substantial improvements in respiratory functions have been reported in an internet survey of 201 smokers who switched to vaping [38]. These improvements are likely attributable to cessation of c-cigarettes rather than the use of e-cigarettes.

#### Harmful effects

**Fig. 4:**
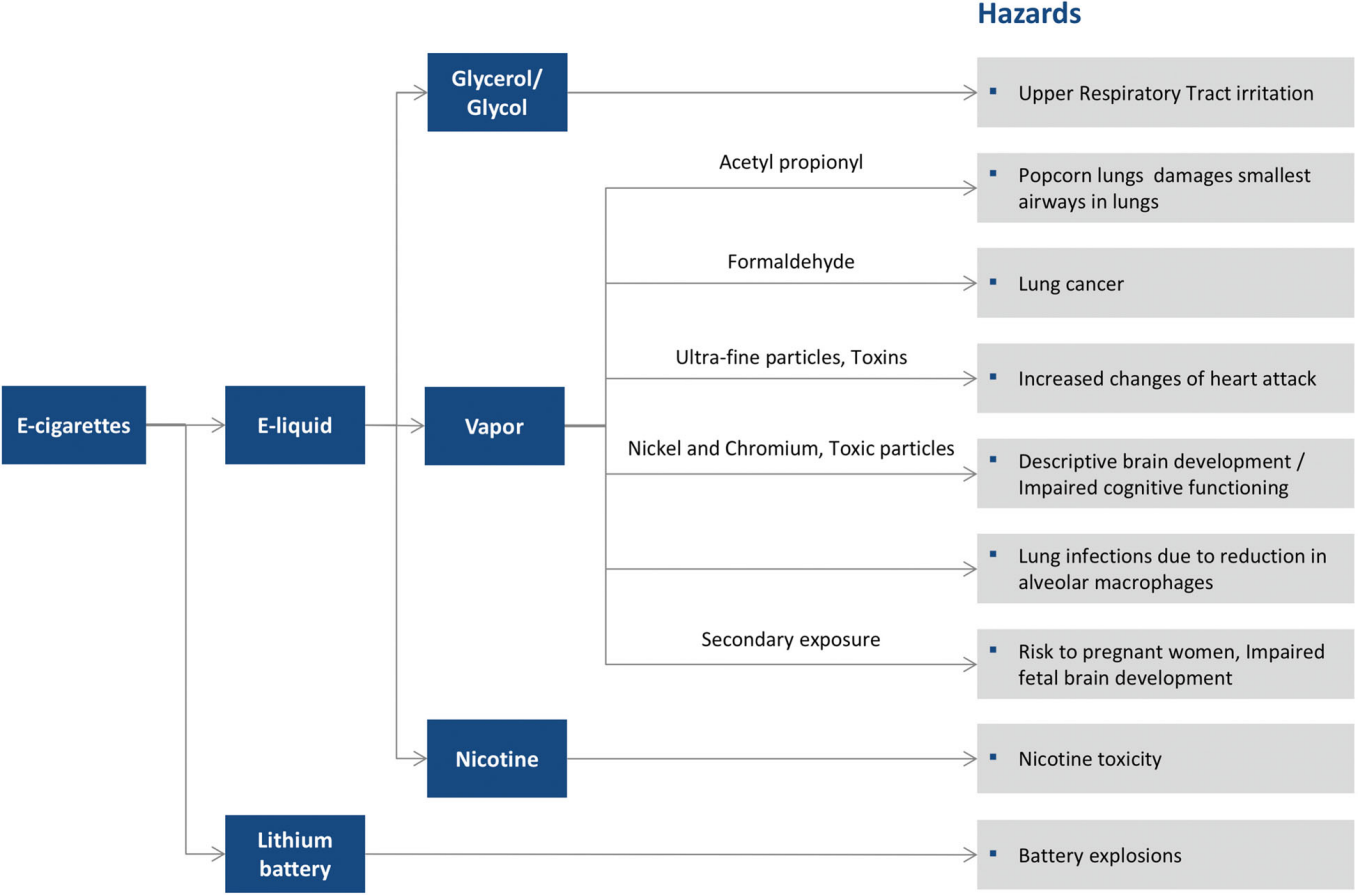
Health hazards of e-cigarette use

The various health hazards associated with the use of e-cigarettes are summarized in Fig. 4.

#### Cardiovascular system

Smoking e-cigarettes daily doubles the risk of heart attack according to research performed by the Center for Tobacco Research and Educational Center at University of California San Francisco (UCSF) in 2018 [39]. This study provided the first evidence of a substantial human health impact of electronic nicotine devices. This new study with nearly 70,000 people found that the heightened risk of heart attack for e-cigarette users is similar to one of the main effects of c-cigarettes. Together, in those who use both c- and e-cigarettes daily, the odds increase five times.

#### Nervous system

Most teens who use e-cigarettes containing nicotine do not know that the drug can be damaging to their development. Extensive evidence shows that the adolescent brain is extremely sensitive to the effects of nicotine and that age is inversely related to the overall effect of nicotine on the brain and body. Nicotine can disrupt brain development, interfere in cognitive functioning, and lead to various physical and mental health issues. A recent study sponsored by the National Institute on Drug Abuse reported that the changes caused by e-cigarettes in the brain are similar to those caused by other nicotine sources, such as c-cigarettes or nicotine lozenges [40]. However, whether e-cigarettes cause addiction in the same way as c-cigarettes remains unknown.

#### Respiratory system

The glycol and glycerol vapors used in vapes are known to cause upper respiratory system irritation. Moreover, acute inhalation of nicotine can cause dizziness, nausea, and/or vomiting. Callahan-Lyon et al. evaluated the particulate matter emissions from e- and c-cigarettes and found that the particulate emissions from e-cigarettes slightly exceeded the World Health Organization’s (WHO) air quality guidelines but were 15 times lower than the emissions from c-cigarettes [32].

#### Immune system

A team of researchers at North Carolina Chapel Hill found that smoking e-cigarettes had a larger effect on human body immunity than traditional c-cigarettes [41]. Pushalkar et al. [3], evaluated saliva of e-cigarette users for changes in oral microbiome and reported significantly altered beta-diversity in (*p* = 0.006) when compared with never or tobacco cigarette smokers. The abundance of *Porphyromonas* and *Veillonella* (*p* = 0.008) was higher among vapers. They further analysed the saliva for host immune response and found high levels of Interleukins (IL)-6 and -1β in e-cigarette users as compared with non-users. E-cigarette aerosol can induce hypoxic condition and induce oxidative stress which can make epithelial cell more susceptible for infection. Pushalkar et al. [3] using in vitro infection model of premalignant Leuk-1 and malignant cell lines exposed to e-cigarette aerosol were more susceptible for infection when exposed to oral pathogens. *P. gingivalis* and *Fusobacterium nucleatum* resulted in. Therefore, people who vape may have elevated inflammatory responses and are more susceptible to infections [3].

#### Reproductive system

The New York Langone group discovered that smoking e-cigarettes might cause potential issues in the reproductive health of individuals, including reducing sperm count in male smokers [42]. According to Centers for Disease Control (CDC) high amounts of nicotine in e-cigarettes are also harmful for fetal brain development if used during pregnancy [43].

#### Exposure risks

Nicotine is readily absorbed from the skin, mucous membranes, airways, and gastrointestinal tract. Toxic reactions are associated with dermal nicotine exposure after spills of nicotine-containing liquids or occupational contact with tobacco leaves [44]. These can result in adverse health effects, such as seizures, anoxic brain injury, and/or lactic acidosis. E-cigarettes pose a poisoning threat to the user and to nonusers due to the availability of the high nicotine content in the cartridges [45]. Use of e-cigarettes in indoor environments may expose nonusers to a high nicotine and aerosol particle levels. Moreover, there is much evidence that drinking alcohol and e-liquid can be fatal [46, 47].

The nicotine from the aerosol can remain on surfaces for weeks to months and may react with ambient nitrous acid (which comes from gas appliances) to form TSNAs that can lead to dermal ingestion or inhalational exposure to carcinogens [48, 49]. The content of the exhaled aerosol may contain different proportions of harmful constituents depending upon the user’s technique or other factors, such as temperature, weather, and airflow [50].

#### Oral cavity

**Fig. 5:**
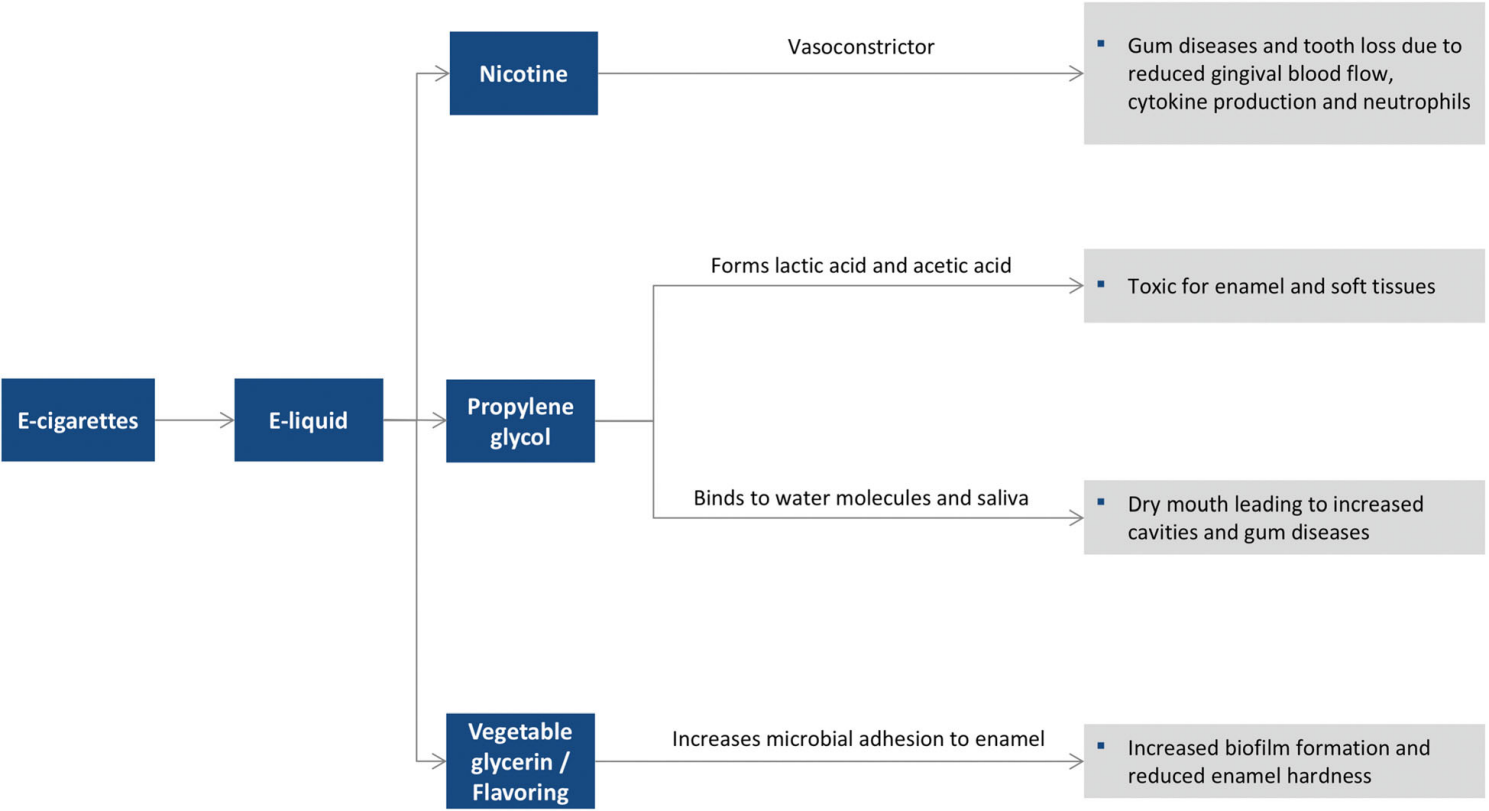
Harmful effects of e-cigarettes on the oral cavity

There have been many reports regarding the adverse effects of e-cigarettes on oral health (Fig. 5). Regulatory agencies caution that e-cig users may absorb higher concentrations of nicotine and other compounds, such as tobacco-specific nitrosamines, aldehydes, metals, and volatile organic compounds**.** Given that nicotine is toxic and addictive, as tobacco alternatives, e-cigs may have similar effects on oral health as traditional cigarettes. The interaction of nicotine and other chemicals in the aerosol produced by e-cigs and the human body occurs first in the oral cavity, where they are expected to be most active and have potent effects on the oral microbiome and oral epithelial cells. Recently we have shown that e-cigarette aerosols alter oral microenvironments and enhance inflammation [1–3]. The major ingredients present in the liquid used in e-cigarettes is propylene glycol (PG), Vegetable glycerin (VG), and Nicotine. PG is a viscous, colorless liquid that possesses a faintly sweet taste and it is breakdown to acetic and lactic acids and other compounds that are all toxic to enamel and soft tissue in oral cavity. VG with other flavoring agents can increase microbial adhesion to enamel and promote biofilm formation. In addition, PG can lead to tissue desiccation resulting in xerostomia (“dry mouth”) promote cavities, gum disease, and other oral health issues. VG and other flavorings agents can decrease enamel hardness. The viscosity of e-liquid enhances the colonization of *Streptococcus mutans* and lead to rampant tooth decay [51].

Nicotine is a well-known vasoconstrictor and it reduces gingival blood flow and affects cytokine production, neutrophil count, and immune cell function, thus increasing the chance of gum disease development and tooth loss [3]. Although the percentage of nicotine is much lower (0.3–1.8%) than that in traditional tobacco products, one electronic cartridge (200–400 puffs) is equivalent to smoking two to three packs of regular cigarettes. E-cigarette aerosol exposure makes epithelial cells susceptible to infection. E-cigarettes further modulates the oral microbiome to increase the abundance of oral pathobionts, alters host response, and promotes inflammation [3].

#### Other hazards

One of the major component of e-cigarettes is the heating device. There are reports that explosion of the lithium batteries used in e-cigarettes can be serious and can cause oral tissue disfigurement. Depending upon the quality of battery and frequent use of the device the battery can be overheated and cause serious injuries. These explosions are usually attributed to improper charging of the device and no internal safety mechanism [52, 53]. Thombs et al., reported that the number of vape explosions in the U.S. is underestimated [52]. In their study they estimated 2035 e-cigarette explosions and burn-related injuries in the US which is more than 40 times the initial estimate by the US government [52, 53].

## Conclusion

Although we have summarized some benefits of e-cigarettes over c-cigarettes, whether e-cigarettes are a beneficial aid for smokers who want to switch from c-cigarettes or whether they have long-term effects on the body are still open questions. The case of Sottera et al. challenged the Food and Drug Administration (FDA) determination of e-cigarettes as unapproved drug/device combinations [54]. The court held that e-cigarettes can be regulated as tobacco products and are not considered drugs or devices unless marketed for therapeutic purposes. This regulation is in contrast to tightly controlled nicotine replacement therapies, such as nicotine patches and inhalers, which are regulated as therapies to promote tobacco cessation.

According to previous studies, the number of adolescents who reported smoking cigarettes in the past month decreased from 28% to only 5% from 1996 to 2018 [55]*.* However, the number of adolescents who had used an e-cigarette in the past 30 days has increased from 1.5% in 2010 to 26.7% in 2018 [1]. These figures show an inverse trend between the growths of e- versus c-cigarette smokers at present among the young adolescent population. Recent reports from the CDC also indicated that the percentage of U.S. middle and high school students who use e-cigs more than doubled. With their increasing popularity, the projected increase in e-cig use over the next few years is of great concern. The increase in adolescent e-cigarette use described above highlights ways that the emergence of e-cigarettes in the market has reversed the years of efforts toward tobacco control.

E-cigarette users may perceive the device as an effective alternative to traditional tobacco smoking, but given the addictive nature of nicotine, which is the main substance in e-cigarettes; these devices eventually lead to a new type of addiction. Limited studies have indicated that e-cigarettes use can have adverse effect on human health. At this stage, since the long-term benefits and risks associated with smoking e-cigarettes have not been studied in detail, it is very difficult to draw conclusions about these new nicotine delivery systems. On the other hand switching to e-cigarettes use can also reduce the consumption of harmful carcinogens which are present in c- cigarettes. Hence, more long-term studies are needed to understand the implication of e-cigarettes in US and world health policies.

## Abbreviations

ENDS: Electronic nicotine delivery systems
TSNA’s: Tobacco-specific nitrosamines
PAH: Polycyclic aromatic hydrocarbons
NCHS: National Center for Health Statistics
NYTS: National Youth Tobacco Survey
AAP: American Academy Pediatrics
UCSF: University of California, San Francisco
WHO: World Health Organization
PG: Propylene glycol
VG: Vegetable glycerin
FDA: Food & Drug Administration
